# Self-evaluated ethical competence of a practicing physiotherapist: a national study in Finland

**DOI:** 10.1186/s12910-020-00469-3

**Published:** 2020-05-29

**Authors:** Kati KULJU, Riitta SUHONEN, Pauli PUUKKA, Anna TOLVANEN, Helena LEINO-KILPI

**Affiliations:** 1 Department of Nursing Science, University of Turku, FI-20014 Turku, Finland; 2 Department of Nursing Science/ Turku University Hospital and City of Turku, Welfare Division, University of Turku, Turku, Finland; 3 National Institute for Health and Welfare, Turku, Finland; 4 Department of Nursing Science, Turku University Hospital, University of Turku, Turku, Finland

**Keywords:** Ethical competence, Physiotherapy, Ethical awareness, Character strength, Self-evaluation, PECET

## Abstract

**Background:**

Patients have the right to equal, respectful treatment. Nowadays, one third of patient complaints concern health care staff’s behavior towards patients. Ethically safe care requires ethical competence, which has been addressed as a core competence in physiotherapy. It has been defined in terms of character strength, ethical awareness, moral judgment skills in decision-making, and willingness to do good. The purpose of this study was to analyze the ethical competence of practicing physiotherapists.

**Method:**

A self-evaluation instrument (Physiotherapist’s Ethical Competence Evaluation Tool) based on an analysis of a concept “ethical competence” was constructed in 2016 and physiotherapists (*n* = 839), working in public health services or private practice responded to the questionnaire.

**Results:**

Based on the results, most of the physiotherapists evaluated themselves highly ethically competent in all areas of ethical competence, subscales being Strength, Awareness, Skills and Will. Willingness to do good was evaluated as highest, while character strength, including the strength to support ethical processes and speak on behalf of the patient, was evaluated the lowest. Physiotherapists most commonly consult a colleague when encountering an ethical problem. Other methods for problem solving are not very familiar, neither are the international or national ethical codes of conduct.

**Conclusions:**

This was the first attempt to assess all aspects of ethical competence empirically in a clinical environment in physiotherapy, using a novel self-evaluation instrument. Even if physiotherapists evaluate themselves as competent in ethics, further exploration is needed for ethical awareness. Also the patients’ viewpoints about ethically competent care should be considered, to better ensure ethical safety of the patient.

## Background

Ethically safe care is a central goal of health care worldwide [1]. All interaction with patients should be human-oriented, recognizing more clearly an individual patient in the center, to ensure dignity and respect in care [2]. This requires ethical competence of a professional and can be acquired through educational interventions [3, 4]. Ethical competence is an important, foundational aspect of health care professionals’ competence. It is considered as a part of professional competence [2], about being honest and loyal to patients [5], requiring abilities of character, action and knowledge [6]. In health care the concept has been defined in many ways - no consensus on the definition can be found in the literature. Concept analysis of ethical competence [7] defines the concept in terms of character strength, ethical awareness, moral judgment skills, and willingness to do good. Ethical competence needs support from the organization, and at the personal level emerges from experience, knowledge and communication. It results in positive outcomes for the patient, professional and society [7]. An integrative literature review [8] states several dimensions of ethical competence, many of them corresponding to those in the previous concept analysis, e.g. sensitivity as a part of ethical awareness and reflection included in moral judgment skills [8].

Physiotherapists as autonomous practitioners, and often working in a multidisciplinary health care environment, also encounter unique and complex ethical challenges. These concern e.g. incompatibility of available resources and patient’s needs [3, 9, 10], unethical behavior of physiotherapists or other professionals and realization of patients’ autonomy [3, 10]. Also close physical and emotional relationship between the patient and the physiotherapist creates specific ethical issues [11, 12], such as how to maintain a professional proximity in the close and very often continued relationship. In addition, ethical issues have raised questions about access to physiotherapy [11, 13] and asymmetrical power between the physiotherapist and the patient [12].

Physiotherapists need skills in clarifying their ethical values and professional moral obligations and in making decisions which are in the best interests of their patients [14, 15]. In the European Qualifications Framework, by the European Commission (2008), ethical competence is described as an integral part of knowledge, skills and competence, and as essential for the development of responsibility and autonomy [16]. The WHO Global Competency Model (2012) states core qualities that are related to ethical competence: active listening, responsibility for one’s own work, ability to identify conflicts, respecting others’ individuality, acting confidentially and according to the ethical and legal framework and personal values [17]. Important is being present, empathetic and supportive and having a reciprocal relationship with patients [18]. Among the core competences in physiotherapy defined in a Finnish survey/Delphi study [19] the ethical aspects were addressed as important and especially ethical sensitivity will be emphasized in the future.

Health care educators, leaders and researchers need to give high priority to the development of ethical competence of health professionals [20]. Ethical competence is assumed to be acquired for example through role models and experience [5], through education and experimental learning in multiprofessional groups [21], by case study method, role-playing [22] and ethics simulations [23]. Ethics education increases ethical awareness and the development of reflective and analytical skills [3, 24] and ethical theories should also be considered as good ways to enhance ethical decision-making skills [3]. Clinical ethics consultations and facilitating mentorship between professionals have been regarded as a good way to assist physiotherapists to identify and negotiate the ethical dimensions of their everyday practice [3, 14].

There are some documents that emphasize an essential knowledge base for ethical competence in health care [16, 17, 25]. Physiotherapists have developed and published their own code of ethics [26, 27], which should support the professionals to clarify their obligations towards patient and the public, and the rights of the patient. However, challenging ethical situations occur in everyday practice when working in different settings with a variety of patients having different ages, values and attitudes, backgrounds and health situations. It is also known that professionals are not very familiar with their ethical codes and how to use them in real situations [12, 15, 28]. They rarely use ethical knowledge, e.g. theoretical frameworks to analyze the ethical issues raised in their practice [29].

Recently, care complaints from patients have raised concerns about patients’ treatment – complaints about health care staff’s attitudes and behaviors towards patients are common and discussed in public [30]. Unethical behavior of health care professionals can result in patients’ diminished courage to ask about their treatment and lack of activity in their own care [31], feelings of depersonalization and ignorance [30] and thoughts of somehow deserving bad treatment. The physiotherapist’s opinion about what’s best for the patient can be completely foreign to the patient and in that situation, it is difficult for the patient to make a commitment to care. Patients want to be involved in their own care and decision-making, which fosters active engagement in physiotherapy [28, 32]. Ethically competent good care results in the best possible solutions for the patients and reduced moral distress of a professional, which is also an important aspect when considering well-being at work [5, 33].

As ethical competence is one of the core competencies in physiotherapy, ethical competence should be continuously evaluated [2]. Assessment of this competence is important to be able to offer effective ethics education, achieve ethical knowledge and skills through education [34], as well as for achieving higher ethical competence at the organizational level. Self-evaluation tool for ethical issues could increase physiotherapists’ interest in ethics, helping them to identify strengths and weaknesses that need to be addressed and to develop critical skills for analysis of their own work [35]. A sufficient theoretical base of the concept has made it possible to precede from conceptualization to measuring ethical competence subjectively [36]. A comprehensive instrument which could measure all the aspects of ethical competence in the physiotherapy context has been lacking. Some instruments have been used to measure mainly parts of ethical competence. In previous physiotherapy research, ethical awareness has been studied as a constitutional aspect in ethical judgment skills (Moral Sensitivity Questionnaire – Revised; Measuring Instrument for Ethical Sensitivity in the Therapeutic Sciences )[10, 37] and also ethical judgment skills have been analyzed in physiotherapy (Defining Issues test) [38].

### The aim of the study

The aim of the study was to evaluate ethical competence of practicing physiotherapists by using a novel self-evaluation tool. In this tool, the dimensions under investigation concerned ethical awareness, character strength and courage, willingness and skills in decision-making. In the future, this tool will be offered to work as a checklist for teachers, supervisors as well as for students and physiotherapists themselves to think and evaluate the skills to identify ethical problems and ethical judgment skills. This study aims to answer the following research questions:

What is the self-evaluated level of ethical competence of physiotherapists? What demographics are associated with the self-evaluated level of ethical competence?

## Methods

A descriptive and correlational study design was used by a cross-sectional questionnaire survey via Webropol 2.0 in spring–autumn 2016. A total sampling from the Finnish Association of Physiotherapists’ membership register was used to maximize variability in age, length of work experience and practice setting. Physiotherapists who had retired or were off work for other reasons (e.g. maternity leave) were excluded. A total sample of 5719 physiotherapists working in various settings (outpatient and inpatient physiotherapy facilities, in private practice) and covering the whole of Finland were invited in this study. A total of 839 valid, completed questionnaires were received via Webropol (response rate 15%).

### Survey instrument

A novel self-evaluation instrument based on the concept analysis [7] and a literature review was used, including a cover letter. The Physiotherapist’s Ethical Competence Evaluation Tool (PECET) is a self-administered, mainly structured questionnaire including two sections: (A) demographic data and background information about ethical knowledge and methods used for ethical decision-making, and (B) self-evaluation of ethical competence designed as a 4-point Likert-type scale consisting of 59 items representing the phenomenon of ethical competence and answering the question “I feel I succeed in this area of ethical competence”, the anchors being 4 = ‘excellent’ and 1 = ‘not at all’. Section B consists of four subscales according to the attributes defined in the concept analysis [7]. The attributes defining ethical competence were repeatedly presented by seven different authors in the reviewed literature. They all illustrate the professional’s personal characteristics: 1) Character strength (12 items, e.g. “I have strength to work as an advocate in client matters. I have strength to work as the client needs, even if it is inconsistent with my own values.”), 2) Awareness (17 items e.g. “I listen to the client. I have sensitivity to identify an ethical dilemma in a situation.”), 3) Skills (17 items e.g. “I know the ethical codes guiding my work. My work is evidence-based.”) and 4) Will (13 items e.g. “I want to act according to the ethical guidelines. I want the patient’s best in all situations.”). (Table 1.)

**Table 1:** Items representing the phenomenon of ethical competence in the questionnaire (PECET)

Subscales	Abbreviated items
**Strength**	1. promote the best of the client 2. act as an advocate of the client 3. work in cooperation with various professional groups 4. act independently 5. take care of own well-being 6. work according to client needs, even if it conflicts with own values 7. work according to client needs, even if it conflicts with organization’s values 8. have the courage to discuss difficult topics 9. support a colleague 10. be brave by nature 11. take the responsibility that the client gets good care despite of insufficient resources 12. take responsibility for the actions
**Awareness**	1. succeed in interaction with client 2. listen to the client 3. recognize the ethical problem 4. recognize the needs of client 5. respect the obligation of secrecy 6. can settle in client’s position 7. take into account client’s social background 8. take into account client’s cultural background 9. take into account client’s opinion 10. respect client’s dignity 11. respect client’s individuality 12. respect client’s self-determination 13. know when doing ethically right 14. aware of own values 15. aware of own attitudes 16. know that working environment can have an impact in ethical decision-making 17. aware of possible pre-emption towards clients
**Skills**	1. professional activity is guided by the ethics guidelines 2. know laws governing professional activity 3. client understands the purpose of therapy 4. client understands the possible consequences of therapy 5. ask client’s informed consent 6. use experts in ethical problem solving 7. use literature in ethical problem solving 8. use support from colleagues 9. act as a responsible expert in work by keeping track of new knowledge 10. recognize limits as a professional 11. work in multiprofessional cooperation in accordance with ethical principles 12. express myself clearly 13. identify the ethical conflict 14. work evidence-based 15. justify therapy choices 16. identify the need to educate more in ethics issues 17. decide on the therapeutic content together with the client
**Will**	1. act according to ethical values 2. treat clients equally 3. promote the client’s best 4. tell the truth to the client 5. act confidentially 6. act according to what I think is right 7. commit to providing high quality care 8. work evidence-based ​ 9. get educated in ethics 10. act evidence-based 11. work in multiprofessional cooperation in accordance with ethical principles 12. justify therapy choices 13. decide on the therapeutic content together with the client

When developing the questionnaire, two expert panels were carried out to evaluate the relevance and clarity of the items to enhance the content validity of the questionnaire [39]. Expert panel I consisted of PhD students in Nursing Science (*n* = 16) (health care professionals, teachers, clinical specialists, health care managers, physiotherapy clients) who had expertise in ethics. Expert panel II consisted of practicing physiotherapists, physiotherapy students and physiotherapy teachers (*n* = 7). Revision of the items was carried out after the panels. A pilot study was conducted with physiotherapy students (*n* = 12) and practicing physiotherapists (*n* = 15). Only minor technical changes for tenses or redundant wording were made based on the pilot study.

### Data analysis

Data from a structured questionnaire were entered into the SAS 9.1 (SAS Institute INC: Cary, NC, USA) statistical software in order to undertake descriptive and inferential statistics. Frequencies, percentages, means and standard deviations (SDs) were used to describe the data. The sum variables were calculated based on the theoretical construct and the internal consistency of items of Section B (Character strength, Awareness, Skills and Will) was measured using Cronbach’s alpha coefficient [40]. Cronbach’s alpha value of 0.70 has been recommended as the lowest acceptable coefficient for a new instrument [39]. The differences between the four sum variables were analyzed by repeated measures analysis of variance. The Tukey-Kramer adjustment for multiple comparisons was used. The correlation between age and working experience and sum variables was examined using Pearson’s correlation test. T-test was used to explore the association between dichotomous variables (e.g. gender, further ethics education, participation of ethics committees etc.) and sum variables. In case of variables with more than two categories (e.g. respondent’s perception of the stage in ethical competence), an analysis of variance was used to test the association between background variables and sum variables of the four subscales in ethical competence (section B). The level of significance was defined as *p* < 0.05.

### Ethical considerations

The University Ethics Committee approved this study. The Finnish Association of Physiotherapists gave the permission for data collection. The respondents were given written information about the aim of the study and informed that answering the questionnaire was considered as informed consent to participate the study. Participation was voluntary. The anonymity of the subjects and confidentiality were considered and protected by treating the data anonymously and confidentially.

## Results

### Respondent characteristics

The physiotherapists’ (*n* = 839) mean age was 45 years (range = 22–70), and the majority of them (90%) were women. The distribution in gender between women and men is the same as member structure of the Finnish Association of Physiotherapists (FAP, 2016). The mean length of working experience was 18 years (range = 0–45 years) (Table 2). The respondents’ current job included diverse areas in neurological, musculoskeletal, pediatric, mental health, geriatric and occupational rehabilitation in inpatient and outpatient care, both in public and in private sectors. All respondents (*n* = 839) had a main degree in physiotherapy either at polytechnic / university of applied sciences or at college level. Some of them (*n* = 81) had also educated themselves further at higher level (e.g. Master in Health Sciences, PhD).

**Table 2:** Respondents’ demographic data (*n* = 839)

	n	%	Mean	Median	SD	Min	Max
Age (years)	839		44.53	46.00	11.57	22	70
Working experience (years)	839		18.10	18.00	11.79	0	45
Gender							
Male	86	10					
Female	753	90					
Education	839						
Polytechnic	414	49					
College level	385	46					
Other^a^	40	5					
Work place	819						
Public sector	392	48					
Private sector	397	48					
Other^b^	30	4					
Encountering of ethical problems at work	833						
Yes	611	73					
No	222	27					
Frequency of encountering ethical problems	614						
Daily	24	4					
Weekly	122	20					
Monthly	175	29					
Rarely	293	48					
Continuing education in ethics after graduation	838						
Yes	166	20					
No	672	80					
Team / committee work in ethics	832						
Yes	24	3					
No	808	97					

SD: standard deviation.

^a^only the highest degree reported (MSc, PhD)

^b^unemployed, researcher, teacher

The respondents used different methods in ethical problem solving and decision-making. When encountering an ethical challenge, physiotherapists mostly consulted a colleague (93% of the respondents). Also discussions in groups (69%) and use of ethics literature (38%) were rather common methods to ease ethical problem solving. Only 8% of the respondents had consulted a specialist in ethics, ethical committees’ help had been needed by 11% of the respondents and theories of ethical decision-making used by 12% of the respondents.

### Self-evaluation of ethical competence

In section B of the constructed instrument PECET, physiotherapists evaluated themselves in four subscales of ethical competence consistent with the theoretical construct of the phenomenon [7]: Character strength, ethical awareness, moral judgment skills and willingness to do good (Table 1). Cronbach’s alphas were calculated to evaluate the internal consistency of the total scale (0.95) and its sum variables (0.76–0.90).

The respondents evaluated themselves highly ethically competent in all subscales of ethical competence. Willingness to do good, to act ethically, was evaluated as highest, while character strength including the ability and strength to support ethical processes and speak on behalf of the patient, was evaluated lowest. All differences between the four subscales were significant (adjusted *p* = 0.028 or less). (Table 3.)

**Table 3:** Sum variables in self-evaluated ethical competence

Variable	n	Mean 1)	SD	Cronbach’s Alpha
Char Strength	834	3.23	0.32	0.76
Awareness	835	3.45	0.33	0.89
Skills	830	3.26	0.39	0.90
Will	830	3.56	0.34	0.88
PECET total	823	3.38	0.29	0.95

1) Scale 1 = not at all, 4 = excellent.

The older (*r* = 0.174, *p* < .0001) and the more experienced (*r* = 0.135, *p* < .0001) the respondent was, the higher self-estimated value was in sum variable Skills.

The connections between dichotomous variables gender (Male/Female), further ethics education (Yes/No), participation of ethics committees (teams; Yes/No), encountering ethical problems at work (Yes/No) and sum variables were detected (Table 4; T-test). In total, participating in further ethics education and ethics committees was associated with higher self-perceived ethical competence in all areas. In addition, female physiotherapists considered themselves more competent than men in all areas. Participating in ethical committees was not common, but the differences were clear. Respondents who reported that they had not encountered ethical problems in their work, considered themselves as competent compared to those who had encountered ethical challenges in their work.

**Table 4:** The association between dichotomous variables gender, further ethics education, participation of ethics committees, encountering ethical problems at work and sum variables

			Character strength		Awareness		Skills		Will		PECET Total	
		n	Mean	SD	Mean	SD	Mean	SD	Mean	SD	Mean	SD
Gender												
	F	738	3.24	0.32	3.46	0.33	3.27	0.39	3.58	0.34	3.39	0.29
	M	85	3.14	0.29	3.36	0.31	3.12	0.34	3.44	0.36	3.27	0.28
	p^a^		0.0086		0.0093		0.0007		0.0003		0.0003	
Further ethics education												
	Yes	165	3.31	0.33	3.50	0.34	3.40	0.38	3.63	0.32	3.46	0.30
	No	668	3.20	0.31	3.44	0.32	3.22	0.38	3.55	0.34	3.35	0.29
	p^a^		<.0001		0.0305		<.0001		0.0055		<.0001	
Ethics committees												
	Yes	24	3.50	0.30	3.72	0.25	3.56	0.32	3.76	0.19	3.64	0.22
	No	803	3.21	0.32	3.44	0.32	3.25	0.38	3.56	0.34	3.37	0.29
	p^a^		<.0001		<.0001		<.0001		<.0001		<.0001	
Encountering ethical problems												
	Yes	608	3.24	0.32	3.45	0.32	3.26	0.38	3.58	0.32	3.38	0.29
	No	220	3.20	0.32	3.45	0.34	3.25	0.39	3.53	0.38	3.36	0.31
	p^a^		0.1186		0.9775		0.6851		0.1426		0.4735	

^a^T-test

The respondents’ self-estimated knowledge of different documents essential for ethical competence varied quite much. Overall, the respondents knew the Finnish Act on the Status and Rights of Patients [41] quite well, the ethical principles of World Confederation for Physical Therapy were known poorly [26]. The Finnish Association of Physiotherapists has their own codes of ethics [27]. Those codes are known excellently by 13% of the respondents, while 30% of the respondents know the Finnish codes poorly or not at all. Those who reported excellent or fairly good knowledge of the documents evaluated themselves also more ethically competent in all areas of ethical competence compared to those reporting poor or no knowledge of the documents. The results were statistically significant.(Table 5.)

**Table 5:** Knowledge of the different key documents and ethical problem-solving methods and its connection to self-evaluated ethical competence

		Character strength		Awareness		Skills		Will		Total	
Document/ Method	n	Mean	SD	Mean	SD	Mean	SD	Mean	SD	Mean	SD
FAP ^a^											
Excellent	109	3.403	0.296	3.640	0.278	3.572	0.319	3.715	0.271	3.585	0.244
Fairly well	474	3.241	0.313	3.462	0.316	3.280	0.363	3.590	0.332	3.396	0.277
Rather poorly	216	3.121	0.295	3.334	0.324	3.086	0.340	3.440	0.346	3.248	0.275
Not at all	36	3.101	0.300	3.381	0.311	2.993	0.415	3.493	0.349	3.238	0.271
p		<.0001		<.0001		<.0001		<.0001		<.0001	
WCPT ^b^											
Excellent	20	3.625	0.198	3.743	0.239	3.794	0.209	3.903	0.125	3.763	0.140
Fairly well	179	3.294	0.336	3.544	0.327	3.417	0.347	3.649	0.322	3.481	0.285
Rather poorly	439	3.210	0.291	3.416	0.305	3.219	0.359	3.544	0.329	3.347	0.269
Not at all	195	3.154	0.326	3.404	0.348	3.132	0.401	3.489	0.358	3.230	0.302
p		<.0001		<.0001		<.0001		<.0001		<.0001	
ETENE ^c^											
Excellent	53	3.479	0.293	3.608	0.290	3.685	0.316	3.750	0.227	3.665	0.231
Fairly well	372	3.260	0.312	3.464	0.322	3.314	0.358	3.588	0.338	3.408	0.286
Rather poorly	326	3.185	0.307	3.414	0.314	3.173	0.353	3.530	0.337	3.326	0.271
Not at all	79	3.076	0.293	3.357	0.340	3.039	0.409	3.464	0.365	3.238	0.283
p		<.0001		<.0001		<.0001		<.0001		<.0001	
Act on the Status and Rights of Patients [41]											
Excellent	139	3.381	0.313	3.617	0.291	3.552	0.336	3.689	0.286	3.567	0.260
Fairly well	479	3.222	0.305	3.430	0.328	3.242	0.352	3.561	0.335	3.364	0.281
Rather poorly	197	3.135	0.321	3.367	0.304	3.378	0.378	3.486	0.358	3.274	0.277
Not at all	18	3.128	0.212	3.556	0.317	3.436	0.436	3.542	0.403	3.313	0.261
p		<.0001		<.0001		<.0001		<.0001		<.0001	
Personal data act [42]											
Excellent	120	3.391	0.315	3.622	0.301	3.534	0.356	3.679	0.292	3.563	0.273
Fairly well	437	3.235	0.307	3.447	0.328	3.273	0.363	3.581	0.338	3.384	0.284
Rather poorly	236	3.148	0.308	3.369	0.298	3.118	0.358	3.492	0.340	3.282	0.266
Not at all	38	3.110	0.297	3.448	0.344	3.049	0.404	3.487	0.386	3.281	0.292
p		<.0001		<.0001		<.0001		<.0001		<.0001	
Consulting a specialist in ethics											
Excellent	11	3.614	0.323	3.786	0.297	3.818	0.239	3.795	0.211	3.762	0.227
Fairly well	66	3.337	0.313	3.546	0.299	3.460	0.327	3.608	0.323	3.493	0.265
Rather poorly	236	3.217	0.305	3.425	0.322	3.246	0.334	3.560	0.335	3.364	0.275
Not at all	333	3.189	0.304	3.421	0.322	3.144	0.382	3.538	0.337	3.324	0.284
p		<.0001		<.0001		<.0001		0.0414		<.0001	
Consulting a colleague											
Excellent	288	3.295	0.313	3.489	0.325	3.326	0.370	3.598	0.322	3.428	0.284
Fairly well	210	3.135	0.282	3.389	0.302	3.145	0.347	3.538	0.335	3.302	0.260
Rather poorly	24	3.024	0.265	3.266	0.326	2.995	0.339	3.356	0.360	3.172	0.273
Not at all	12	3.128	0.453	3.399	0.357	3.015	0.525	3.385	0.414	3.264	0.391
p		<.0001		0.0002		<.0001		0.0008		<.0001	
Theories in ethical decision-making											
Excellent	16	3.483	0.332	3.625	0.368	3.597	0.363	3.748	0.310	3.616	0.297
Fairly well	212	3.308	0.307	3.523	0.332	3.430	0.346	3.643	0.320	3.482	0.279
Rather poorly	307	3.203	0.307	3.425	0.324	3.235	0.359	3.533	0.342	3.350	0.287
Not at all	268	3.187	0.319	3.429	0.316	3.141	0.384	3.545	0.332	3.328	0.278
p		<.0001		0.0033		<.0001		0.0019		<.0001	
Ethics literature											
Excellent	26	3.503	0.270	3.744	0.243	3.707	0.283	3.875	0.161	3.714	0.189
Fairly well	195	3.307	0.300	3.497	0.320	3.399	0.345	3.627	0.296	3.460	0.266
Rather poorly	378	3.198	0.301	3.417	0.330	3.185	0.344	3.531	0.345	3.332	0.281
Not at all	115	3.107	0.322	3.381	0.305	3.021	0.377	3.469	0.354	3.251	0.271
p		<.0001		<.0001		<.0001		<.0001		<.0001	
Ethical committees											
Excellent	34	3.370	0.358	3.623	0.345	3.539	0.394	3.666	0.337	3.558	0.316
Fairly well	134	3.329	0.338	3.527	0.293	3.397	0.318	3.668	0.281	3.483	0.250
Rather poorly	341	3.201	0.300	3.411	0.328	3.216	0.362	3.546	0.346	3.343	0.285
Not at all	204	3.162	0.301	3.412	0.319	3.114	0.388	3.500	0.343	3.301	0.281
p		<.0001		<.0001		<.0001		<.0001		<.0001	
Ethics education											
Excellent	16	3.653	0.224	3.820	0.191	3.843	0.179	3.875	0.143	3.806	0.147
Fairly well	66	3.297	0.316	3.489	0.339	3.413	0.330	3.647	0.299	3.466	0.277
Rather poorly	364	3.229	0.314	3.430	0.321	3.246	0.352	3.559	0.344	3.366	0.280
Not at all	275	3.178	0.306	3.434	0.316	3.150	0.391	3.532	0.336	3.327	0.282
p		<.0001		<.0001		<.0001		0.0002		<.0001	
Group discussion											
Excellent	113	3.345	0.324	3.563	0.311	3.436	0.352	3.662	0.285	3.505	0.271
Fairly well	259	3.202	0.300	3.429	0.305	3.228	0.341	3.575	0.324	3.358	0.262
Rather poorly	161	3.192	0.318	3.390	0.335	3.146	0.353	3.489	0.364	3.307	0.286
Not at all	87	3.126	0.277	3.403	0.313	3.034	0.408	3.498	0.348	3.269	0.282
p		<.0001		<.0001		<.0001		<.0001		<.0001	

^a^Finnish Association of Physiotherapists. 2014. Codes of Ethics [27]

^b^World Confederation for Physical Therapy. 2011. Ethical Principles [26]

^c^The National Advisory Board on Social Welfare and Health Care Ethics. 2001. Shared values in health care, common goals and principles: Principles of Health Care Ethics [43]

Generally, the respondents were not very familiar with different methods for ethical problem solving and decision-making. The best (excellently or fairly well) were known consulting a colleague (93%) and discussions in groups (60%), while a minority of the respondents knew excellently or fairly well ethical theories (28%) and literature (31%) or other methods that could be used to enhance ethical decision-making. Use of ethical committees or ethics specialists were known rather poorly or not at all by a majority of the respondents. Those who reported excellent or fairly good knowledge of the methods evaluated themselves also more ethically competent in all areas of ethical competence compared to those reporting poor or no knowledge of the methods. The results were statistically significant. (Table 5.)

As illustrated in the background information of the respondents, only 12% of respondents had used ethical theories to ease ethical decision-making. These respondents also evaluated statistically significantly higher their ethical competence in the Skills sub-variable (*p* = 0.0049). The 11% who had needed help from ethical committees, also had higher values in the Skills sub variable (*p* = 0.0002), but also in the Character strength sub-variable (*p* = 0.0001). One third of the respondents (38%) had used ethical literature to help ethical problem solving. These respondents also evaluated statistically significantly higher ethical competence in sub-variables Character strength (*p* = <.0001), Skills (*p* = <.0001) and Will (*p* = 0.0009). Overall, the better the respondents knew different ways in ethical problem-solving and decision-making (consulting an ethics specialist, consulting a colleague, group work, ethics literature, theories, committees, further ethics education), the higher they evaluated themselves in ethical competence. This connection was statistically significant in total PECET (*p* = <.0001).

## Discussion

The aim of this study was to evaluate the ethical competence of a practicing physiotherapist by using a novel self-evaluation tool in Finland. The total level of ethical competence PECET_total_ (Character strength, Awareness, Skills and Will) was assessed quite high, at 3.58 (anchors being 1 = not at all, 4 = excellent). Age and length of working experience correlated with ethical judgment skills. The older and more experienced the respondent was, the higher the self-estimated value was in the sum variable Skills. This finding supports the previous study of Höglund, Eriksson, and Helgesson [5] as they state that ethical competence can be acquired through experience. Willingness to do good, to act ethically towards beneficence of the patient, was evaluated as highest. Also Praestegaard and Gard [12, 28] have highlighted that physiotherapists desire to work for the patient’s well-being.

It seems that physiotherapists still have different abilities to recognize ethical issues: almost one third of the respondents reported that they have never encountered ethical problems in their practice. Interestingly, these respondents considered themselves as ethically competent as compared to those who have encountered ethical challenges in their work. This comes to a question of ethical awareness. If the ethical aspects of a situation are not recognized, it is difficult to address any ethical problem [37] – physiotherapists need to be challenged to practice identifying ethical issues and being ethically conscious in all interaction with the patient [12, 28].

Physiotherapists are not very familiar with different methods and aids that could be used in ethical judgment and decision-making, such as ethics rounds, ethics committees, ethical theories or ethics literature. This finding is consistent with previous studies [12, 29] which state that physiotherapists rarely use ethical knowledge to analyze ethical issues. Knowledge of the different methods, but also knowledge of the different documents related to ethical decision-making, are positively associated with higher self-estimated ethical competence. This finding follows the previous study of Delany [14] and is also consistent with the concept analysis [7] which states the knowledge of ethics to be the prerequisite of ethical competence. Furthermore, further ethics education is positively connected to physiotherapist’s self-estimated ethical competence. That result is consistent with previous research which notes that ethical competence can be acquired through education [4, 21, 24]. Taking part in ethics education after graduation is still very uncommon among physiotherapists (20% had participated). To support the ethical competence of physiotherapists, multidisciplinary ethics committees in health care organizations and also ethics consultation and education are available. The possibilities still vary among organizations to get consultation in ethical issues, possibilities for all health care professionals to strengthen their ethical competence, develop their ethical knowledge, awareness and courage and moral judgment skills, in ethics discussion groups, virtual ethics labs or by playing ethics games.

### Strengths and limitations

In the physiotherapy field, this was the first attempt to assess all aspects of ethical competence empirically in a clinical environment and the concept of ethical competence was for the first time operationalized in this extent. The PECET instrument needs further development to be used as a checklist, thought-provoking tool as a part of ethical reflection. Because the survey instrument is a self-evaluation tool in nature, the respondents are demanded to acquire and use self-reflection skills to analyze their knowledge and action in quite a difficult topic. How they use these skills is a question of validity and raises a question about what actually is being measured with the self-evaluation tool. Ethics in clinical practice can be very complicated and choosing how to act depends on how a situation is interpreted. The respondents assessed themselves very positively in all dimensions of ethical competence, even though 27 % reported that they have not encountered ethical problems in their practice. This is a contradictory result that is difficult to justify and raises a question of validity of the novel instrument. Attaching Ethical guidelines for physiotherapists [27] and a definition of ethical problem to the questionnaire would have facilitated answering the questions. Besides ethical awareness, this may also be a question of over assessment of knowledge and skills [35]. However, self-assessment can increase the interest for ethical issues and enhance critical thinking among physiotherapists to analyze their own work, which is an essential component of lifelong learning [35]. The survey instrument used in this study was constructed based on a concept analysis of the concept ethical competence, which formed a theoretically solid ground for the questionnaire. The internal consistency of the constructed instrument was good, for the total scale 0.95 and between the sum variables Strength, Awareness, Skills and Will 0.76–0.90 being acceptable [39].

The number of respondents (*n* = 839) was statistically sufficient, but the response rate (15%) was low representing less than one fifth of all possible respondents and thus, leading to low generalizability of the results. The low response rate may be because the data were collected electronically via Webropol. Also, the topic may be perceived as difficult to think of or not considered as important as hands-on clinical skills. Since the data were collected from the Finnish Physiotherapist Association’s membership register, it can be assumed that the results are nationally representative.

It is worth discussing, if ethical competence can in its wholeness be measured objectively in physiotherapy or generally in health care, using quantitative measures. However, the way physiotherapists evaluate their ethical competence is important as this would likely affect whether they see a need to pay attention to their ethical decisions or educate themselves in ethical issues, even when it does not tell about physiotherapists’ actual capacity to act ethically in a given situation. This article describes the implementation of a novel self-evaluation instrument to measure ethical competence from a physiotherapist’s viewpoint, leaving the patient’s point of view and experiences still incomplete. This needs further consideration.

## Conclusion

This study was the first attempt to evaluate ethical competence in this extent in the context of physiotherapy. The ultimate goal was to enhance physiotherapists’ ethical knowledge and awareness of moral issues and illustrate the role of ethics in physiotherapy to improve the ethical quality of physiotherapy care. Constructing an instrument to be able to self-evaluate these aspects in physiotherapy situations has been a step towards this goal. Physiotherapists consider themselves quite competent in ethics, even if they are not very familiar with ethical codes or methods for ethical problem-solving. The competence to recognize ethical issues in a situation, ethical awareness, needs further exploration, as almost one third of the respondents report they have not encountered ethical challenges in their practice. Both quantitative and qualitative data should be collected to consider the patients’ viewpoint about ethically competent care, to better ensure the ethical safety of the patient. Also, the exploration of the structure of the PECET using confirmatory factor analysis, and Rasch analysis for the item level assessment should be conducted in the future.

## Data Availability

The datasets used and/or analyzed during the current study are available from the corresponding author on reasonable request.

## Abbreviations

ETENE: The national advisory board on social welfare and health care ethics
FAP: Finnish association for physiotherapists
PECET: Physiotherapist’s ethical competence evaluation tool
SD: Standard deviation
WCPT: World confederation for physical therapy
